# The LIM-only protein, LMO4, and the LIM domain-binding protein, LDB1, expression in squamous cell carcinomas of the oral cavity

**DOI:** 10.1038/sj.bjc.6600952

**Published:** 2003-05-13

**Authors:** H Mizunuma, J Miyazawa, K Sanada, K Imai

**Affiliations:** 1 Department of Biochemistry, School of Dentistry, Nippon Dental University, 1-9-20 Fujimi, Chiyoda-ku, Tokyo 102-8159, Japan; 2 Department of Oral Surgery, School of Dentistry, Nippon Dental University, 1-9-20 Fujimi, Chiyoda-ku, Tokyo 102-8159, Japan

**Keywords:** LMO4, LDB1, LIM, carcinoma

## Abstract

Carcinoma cells can lose their epithelial cell characteristics and dedifferentiate into a fibroblast-like cell during progression of a neoplasm. Aberrant expression of oligomeric transcriptional complexes contributes to progression of carcinomas. Although individual transcription factors initiating progression remain unknown, LIM-only protein (LMO) and LIM-domain binding protein (LDB) negatively regulate breast carcinoma cell differentiation. In this study, we investigated the expression of LMO4 and LDB in squamous cell carcinomas of the oral cavity. *LMO4* mRNA was amplified in four of six carcinoma tissues and eight of 12 carcinoma cell lines, and *LDB1* in three carcinoma tissues and 11 cell lines examined. Immunoprecipitation studies revealed that LMO4 and LDB1 interact with each other in the nuclear milieu of the carcinoma cells indicating the presence of an LMO4-LDB1-mediated transcription complex. Both LMO4 and LDB1 proteins were preferentially localised in the nuclei of carcinoma cells at the invasive front and the immunoreactivity was increased in less-differentiated carcinoma tissues (*P*<0.01). Carcinoma cells metastasised to the cervical lymph nodes with increased immunoreactivity compared to the primary site of neoplasm (*P*<0.05). These data suggest that the LMO4–LDB1 complexes may be involved in carcinoma progression possibly through dedifferentiation of squamous carcinoma cells of the oral cavity.

Oral squamous cell carcinoma is the most common neoplasm of the head and neck. Worldwide, the annual incidence of new cases exceeds 300 000. The disease causes great morbidity, and the 5-year survival rate has not improved in more than two decades (Vokes *et al*, 1993; Lippman and Hong, 2001). With few exceptions, carcinomas are derived from single somatic cells and their progeny. Carcinoma cells in the emerging neoplastic clone accumulate within them a series of genetic and/or epigenetic changes that lead to changes in gene activity, and altered phenotypes which are subjected to selection of tumour progression (Ponder, 2001). The generation of cellular diversity in carcinomas frequently involves aberrant expression of transcriptional regulators acting in a combinational manner. Loss of epithelial morphology and acquisition of mesenchymal characteristics, often referred to as the epithelial–mesenchymal transition, are typical for carcinoma cells in dedifferentiation and correlate with tumour progression (Hay, 1995; Birchmeire *et al*, 1996; Thiery, 2002). However, the genetic basis of dedifferentiation and progression of carcinoma cells has not been determined.

The LIM-only (LMO) protein carries two tandemly repeat LIM zinc-binding domains and consists of four members (designated as LMO1–4). The LIM domain has been identified in a variety of nuclear proteins (Sanchez-Garcia and Rabbitts, 1994). It functions primarily as a module for the assembly of protein complexes through protein–protein interactions. The LIM domain does not directly interact with DNA, but acts as an adaptor molecule for transcription factors facilitating assembly of large transcriptional complexes (Breen *et al*, 1998; Jurata *et al*, 1998; Sugihara *et al*, 1998). LMO1 and LMO2 have been shown to specify neuronal and haematopoietic cell lineages in combination with their transcription partners (Hinks *et al*, 1997; Yamada *et al*, 1998; Herblot *et al*, 2000). Misexpression of these genes by chromosomal translocation abrogates proper differentiation of cells and is oncogenic within T cells (Boehm *et al*, 1991; Royer-Pokora *et al*, 1991). Little is known about LMO3, which was discovered on the basis of sequence homology. The most recently identified member, LMO4, shares only 50% homology with the LIM domains of other LMO proteins. The LMO4 gene is widely distributed in embryonic tissues (Kenny *et al*, 1998; Sugihara *et al*, 1998), and involved in negative regulation of breast carcinoma cell differentiation (Visvader *et al*, 2001). LMO4 binds with a high affinity to the LIM domain-binding proteins, LDB1 (CLIM2, NLI1) and LDB2 (CLIM1) (Agulnick *et al*, 1996; Jurata *et al*, 1996; Sugihara *et al*, 1998). LDB proteins bind to transcription factors directly or indirectly mediated through LMO proteins, and then bridge a unique bipartite DNA sequence separated by about one helix turn from each other (Agulnick *et al*, 1996; Jurata *et al*, 1996; Wadman *et al*, 1997; Rabbitts, 1998). Both LMOs and LDBs are widely expressed during development (Kenny *et al*, 1998; Millan *et al*, 1998; Toyama *et al*, 1998; Hermanson *et al*, 1999) and appear to have essential functions in cell proliferation and lineage determination, and oncogenesis (Jurata *et al*, 1998; Milán *et al*, 1998; Rabbitts, 1998; Thaler *et al*, 2002).

Prompted by the observation that LMO4 and LDB1 are expressed in embryonic epithelial tissues (Bach *et al*, 1997; Sugihara *et al*, 1998), we examined the expression pattern of LMO4 and LDBs in squamous carcinoma cells. In contrast to the negligible expression of LDB2, LMO4 and LDB1 were frequently detected in the less-differentiated carcinomas and carcinoma cells at the invasive front, and upregulated in metastasised lymph nodes, suggesting an involvement of the LMO4–LDB1 transcriptional complex in the pathology of carcinoma progression.

## MATERIALS AND METHODS

### Cell lines and tissue samples

Immortalised cell lines established from oral squamous carcinomas (Ca9.22, Ho1N1, HOC313, HSC2, HSC3, HSC4, KOSC2, KOSC3, OSC19, SCCKN, SCCTF, and TSU) were obtained from the Cell Resource Center for Biomedical Research Institute of Development, Aging and Cancer (Tohoku University, Sendai, Japan), Health Science Research Resources Bank (Osaka, Japan) or RIKEN Cell Bank (Tsukuba, Japan), and maintained in 10% fetal bovine serum (FBS)-containing DMEM or RPMI1640 medium (Invitrogen, Grand Island, NY, USA) in a 5% CO_2_ incubator. Normal gingival fibroblasts (GF12) were maintained in 10% FBS containing DMEM for 19 passages (Takahashi *et al*, 1997). HaCaT cells (Boukamp *et al*, 1988), immortalised normal keratinocytes, were grown in DMEM supplemented with 10% FBS.

A total of six oral carcinoma tissues (three well-differentiated carcinomas and three moderately differentiated carcinomas) and three normal oral tissues without a history of head and neck carcinoma were obtained from patients undergoing surgery for carcinoma resection or dental surgery at the Nippon Dental University Hospital, Meikai University Hospital, or Machida City Hospital under the informed consent of the patients.

### Reverse transcription–PCR

Total RNA was isolated from cell lines at 70–85% confluency, oral squamous cell carcinomas, and normal oral tissues using TRIzol reagent (Invitrogen) followed by RNase-free DNase I treatment to eliminate DNA contamination in the sample. After reverse transcription (RT) to a single-stranded cDNA using SuperScript II (Invitrogen) and random hexamer (Invitrogen) at 42°C for 60 min, PCR reaction was performed with gene-specific primer sets for *LMO4* (forward: exon 4; 5′-CGGGAGATCGGTTTCACTAC, reverse: exon 5; 5′-CCAGTGCCCTGCTAATTGTT), *LDB1* (forward: exon 5; 5′-TGCCATGTTGACCATCACTT, reverse: exon 9; 5′-GGC-TGAGGCTGTAGGTCTTG), *LDB2* (forward: exon 1; 5′-TTTCGA-AAAGCAGGCAAGAT, reverse: exon 6; 5′-TCGGGGACTGAGGTTGTAAG), or *GAPDH* (forward: 5′-GTCAGTGGTGGACCAGACCT, reverse: 5′-AGGGGAGATTCAGTGTGGTG) and Taq DNA polymerase (Invitrogen). PCR amplification was performed by running 30 cycles under the following conditions: denatured at 94°C for 40 s, annealed for 40 s at 60°C, and extended at 72°C for 1 min. PCR amplicons were analysed on 2% agarose gels.

### Tissue specimen selection

Incisional or excisional biopsy specimens from 49 patients with oral squamous cell carcinomas were collected from the files of The Kanazawa University Hospital from 1991 to 2000 under informed consent of the patients. Clinical and pathological data were obtained from the patients' medical records and The Kanazawa University Hospital Surgical Pathology files. Clinical and pathologic variables included age, gender, tumour size, tumour location, grade of tumour differentiation, and presence or absence of cervical lymph node metastasis. Control normal tissues (*n*=5) were also obtained at surgery or autopsy from tongue and gingiva of patients without a history of head and neck cancer. These samples were immediately fixed in 10% neutral buffered formalin or periodate–lysine–paraformaldehyde (PLP) solution and embedded in paraffin wax.

### Immunohistochemistry

The haematoxylin and eosin-stained slides from each of the cases were screened by light microscopy. Unstained serial sections (4 *μ*m) were deparaffinised and rehydrated followed by the microwave treatment in 0.01 M sodium citrate buffer, pH 6.0 (500 W). After incubation with normal serum, sections were incubated with rabbit anti-LMO4 (5 *μ*g ml^−1^, Chemicon, Temecula, CA, USA), goat anti-LDB1 (2 *μ*g ml^−1^, Santa Cruz Biotechnology, Santa Cruz, CA, USA) or goat anti-LDB2 (2 *μ*g ml^−1^, Santa Cruz Biotechnology) antibodies for 12 h at 4°C. Biotinylated anti-rabbit or -goat IgG (DAKO, Glostrup, Denmark) was used for secondary antibody followed by incubation with avidin–biotin complexes (Vector Laboratories, Burlingame, CA, USA). The colour was developed with 3,3′-diaminobenzidine tetrahydrochloride (Sigma-Aldrich, St Louis, MI, USA) under the microscope and counterstained with methyl green. To clarify the specificity of the antibody reactivity, primary antibodies were replaced with either nonimmune rabbit or goat IgG (DAKO) at matched protein concentrations. Carcinoma cells with a strong nuclear labelling were determined as positive reactions, but cells with a weak nuclear staining and/or diffuse cytoplasmic staining were not counted as positive. The percentage of positive nuclear staining of LMO4 or LDB1 was evaluated by counting at least 3000 carcinoma cells in randomly selected areas of each specimen at × 40. They were blinded as to the clinicopathological parameters.

### Immunocytochemistry

Human oral squamous carcinoma cell lines (TSU, HOC313, HSC3, and OSC19) were cultured on glass slides (Lab-Tek Chamber II, NUNC, Naperville, IL, USA) in 10% FBS-containing medium and fixed in 1% paraformaldehyde for 3 min at 23°C. The cells were reacted with primary antibodies to LMO4 (10 *μ*g ml^−1^), LDB1 (4 *μ*g ml^−1^), or LDB2 (4 *μ*g ml^−1^) for 16 h at 4°C. Alexa Fluor 546 anti-rabbit or -goat IgG (Molecular Probes, Eugene, OR, USA) was used for secondary antibody. To clarify the specificity of antibody reactivity, incubation with nonimmune rabbit IgG or goat IgG at matched protein concentrations instead of primary antibodies was performed.

### Immunoprecipitation

For detection of endogenous protein binding, crude nuclear extracts (30 *μ*g) of HSC3, TSU, OSC19, or HOC313 cells were prepared by the method described elsewhere (Digman *et al*, 1983; Lee *et al*, 1998), immunoprecipitated with goat anti-LDB1 antibody (2 *μ*g) and protein G-Sepharose (Pharmacia, Wikströms, Sweden), and size fractionated by SDS–PAGE (10% total acrylamide) under reducing condition. After electrotransfer to nitrocellulose membranes, membranes were blocked by 3% bovine serum albumin and incubated with rabbit anti-LMO4 or goat anti-LDB1 antibodies, followed by incubation with biotinylated secondary antibodies. Avidin–biotin complex and 3,3′-diaminobenzidin tetrahydrochloride were used for the colour development.

### Statistical analysis

One-way analysis of variance followed by contrast statements (Scheffe's *F*-test and Fisher's PLSD) was performed to compare LMO4 or LDB1 immunoreactivity with carcinoma differentiation and other clinical and pathological parameters recorded in the text. Statistical analysis of correlation between the immunoreactivity of LMO4 and LDB1 was conducted by simple linear regression and between primary site and metastasised lymph nodes by Wilcoxon's signed-rank test.

## RESULTS

### Expression of LMO4, LDB1, and LDB2 in oral carcinomas

**Figure 1:**
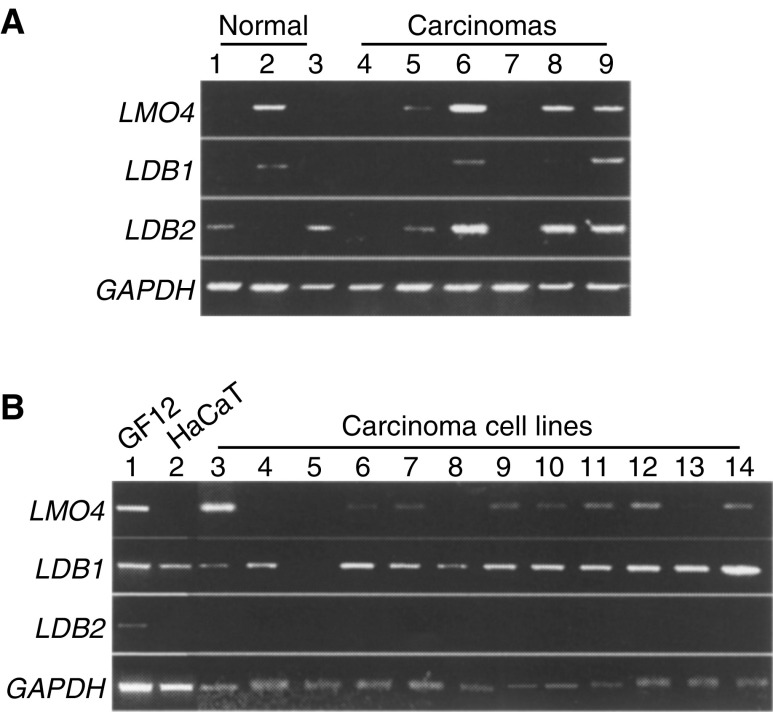
Expression of *LMO4* and *LDBs* mRNA in oral squamous cell carcinomas. (**A**) *LMO4*, *LDB1*, and *LDB2* transcripts are amplified in normal gingiva (lanes 1–3) and carcinoma tissues (lanes 4–9) by RT-PCR. A single 485, 494, or 702 bp fragment was observed by a specific primer set for *LMO4*, *LDB1*, or *LDB2*, respectively. *GAPDH* (395 bp) was included as an internal control. (**B**) Oral squamous carcinoma cell lines were subjected to RT–PCR analysis (lane 3; HOC313, lane 4; TSU, lane 5; HSC3, lane 6; HSC4, lane 7; KOSC2, lane 8; Ho1N1, lane 9; Ca9.22, lane 10; SCCKN, lane 11; KOSC3, lane 12; SCCTF, lane 13; HSC2, lane 14; OSC19). RNA sample isolated from GF12 normal gingival fibroblasts (lane 1) and HaCaT cells (lane 2) were applied as controls.

Expression of *LMO4*, *LDB1*, and *LDB2* in squamous cell carcinomas has not been investigated. We therefore examined the expression pattern of these genes in carcinoma tissues of the oral cavity by RT–PCR (Figure 1A

). Based on the human genome database (http://www.ncbi.nlm.nih.gov/blast/Blast.cgi), primer sets for *LMO4*, *LDB1*, or *LDB2* were designed as spanning one or more introns to prevent amplification of genomic DNA. *LMO4* and *LDB1* exhibited an almost identical pattern of expression. Specific primer sets for each gene amplified a single product; four of six and three of six carcinomas for *LMO4* (485 bp) and *LDB1* (494 bp), respectively. Normal gingiva obtained from patients without a history of head and neck cancer also expressed the genes in one of the three samples. Four carcinomas and two normal samples augmented a single 702 bp fragment of the *LDB2* transcript.

We analysed RNA samples isolated from tissues containing epithelial and mesenchymal components. To remove mesenchymal cell contamination from carcinoma cells, we examined the expression of *LMO4*, *LDB1*, and *LDB2* in 12 different squamous carcinoma cell lines (Figure 1B). *LMO4* was amplified in eight of 12 cell lines and *LDB1* in 11 of 12 carcinoma cell lines. *LMO4* was amplified in GF12 normal fibroblasts and *LDB1* in GF12 and HaCaT cell lines. *LDB2* was only detected in GF12, but not in any of the cell lines of epithelial origin.

### Protein expression of LMO4 and LDB1, and interaction in carcinoma cells

**Figure 2:**
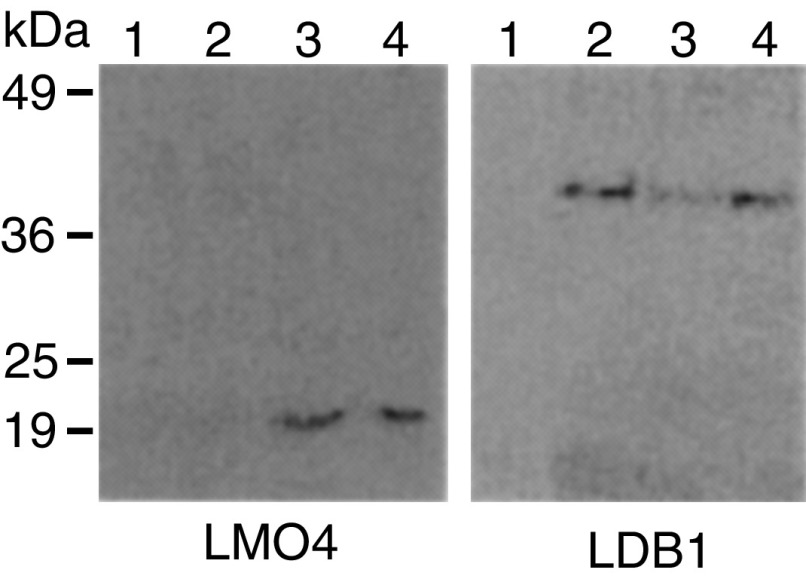
Analysis of protein interaction in squamous carcinoma cell lines. Nuclear extracts from HSC3 (lane 1), TSU (lane 2), OSC19 (lane 3), or HOC313 cells (lane 4) were immunoprecipitated using anti-LDB1 antibody. After SDS–PAGE, immunoblotting was performed using antibodies specific to LMO4 or LDB1.

Since *LMO4* and *LDB1* were expressed in oral carcinomas, we examined the binding between LMO4 and LDB1 proteins by immunoprecipitation. LMO4 was coimmunoprecipitated with an anti-LDB1 antibody in OSC19 and HOC313 cells, which expressed *LMO4* and *LDB1* genes (Figure 2

). The molecular size of LDB1 has not been determined, but is expected to be around *M*_r_ 42 809 from the deduced amino-acid sequence. LDB1 exhibits a single reactive protein band at *M_r_* 40 000. TSU cells, which amplified *LDB1*, but not *LMO4* genes, were immunoprecipitated only by LDB1 protein. As expected, *LMO4*- and *LDB1*-negative HSC3 cells did not react with the antibodies.

**Figure 3:**
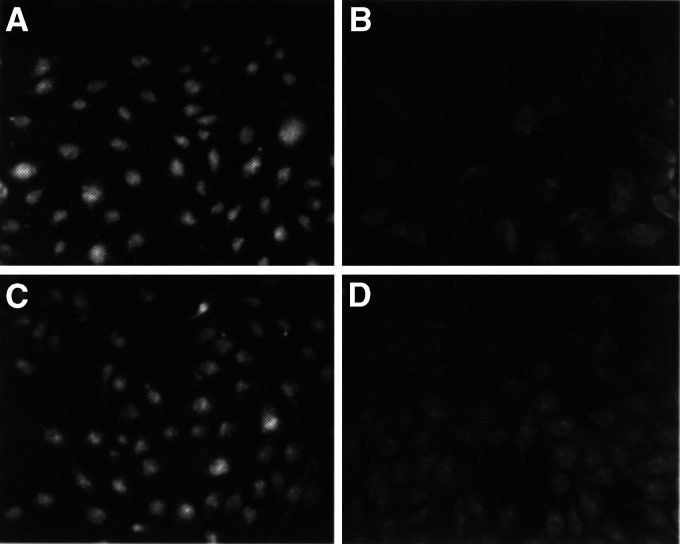
Immunocytochemisty of LMO4 or LDB1 in carcinoma cells. Squamous carcinoma cells cultured on the slide glasses were applied for immunostaining to LMO4 (**A**, **B**) or LDB1 (**C**, **D**). (A, C) HOC313 cells (B, D) HSC3 cells. Bar=50 *μ*m.

Immunocytochemistry stained the nuclei of HOC313 and OSC19 cells for LMO4, OSC19, HOC313, and TSU for LDB1, and none for LDB2 (Figure 3A and C

). HSC3 cells were not stained by antibodies against LMO4 or LDB1 (Figure 3B and D). No staining was observed with nonimmune IgG (data not shown). Specificity of the antibodies used in this study was ascertained by nuclear immunostainings and by the appropriate size of a single-reactive band to the predicted molecular weight. Thus, these data demonstrate that LMO4 and LDB1 established a protein complex in oral squamous carcinoma cells.

### Tissue localisation of LMO4 and LDB1 in oral carcinoma tissues

Clinical and pathological characteristics of the 49 patients with oral squamous cell carcinoma were collected and analysed. The samples were obtained from the tongue (20 cases), gingiva (15 cases), oral floor (six cases), buccal mucosa (five cases), lip (two cases), or maxillal sinus (one case). The patients (21 males and 28 females) ranged from 37 to 92 years of age (mean, 66 years old). Histopathological typing of carcinomas was classified into three groups; well (21 cases), moderately (23 cases), and poorly (five cases) differentiated carcinoma groups. None of the cases represented were classified as squamous carcinomas with a spindle-cell morphology. Lymph node tissues with metastasis of carcinomas were also subjected to the immunohistochemistry procedure (*n*=7).

**Figure 4:**
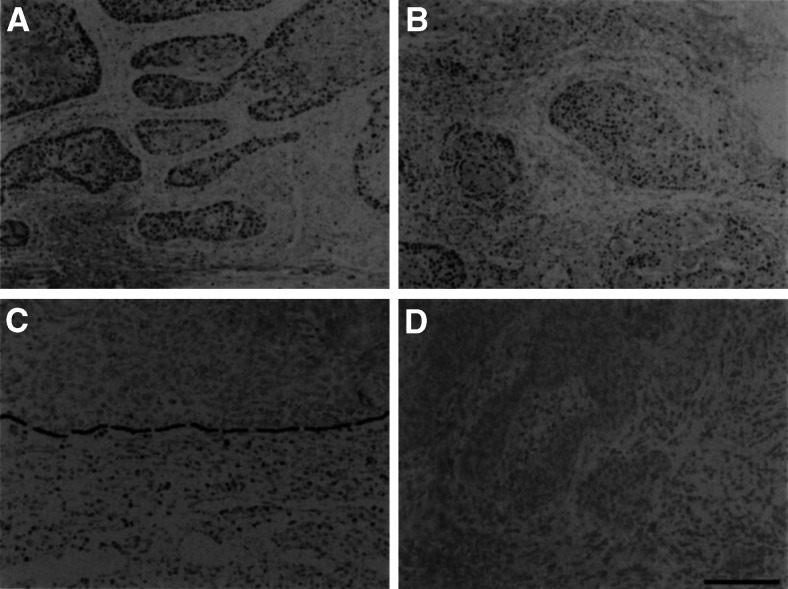
Immunolocalisation of LMO4, LDB1, or LDB2 in carcinoma or normal tissues. LMO4 (**A**) and LDB1 (**B**) were localised to carcinoma cells and stromal cells at the invasive front. Lymphocytes infiltrated into the submucosal layer of normal gingival were immunoreactive to LDB2 staining (**C**). Broken line depicted the borderline between epithelium and mesenchyme. Negative control staining with nonimmune goat IgG was performed instead of primary antibody (**D**). Bar=250 *μ*m (A, B, and D) and 125 *μ*m (C).

We localised LMO4 and LDBs in tissue sections by immunostaining using specific antibodies. Nuclear staining of LMO4 and LDB1 was observed in 30 (61%) and 37 (76%) of 49 carcinomas, respectively. Although minimal reaction of LDB2 immunostaining was noted in inflammatory cells that infiltrated into the carcinoma tissues, carcinoma cells did not react (data not shown). Normal epithelium from gingiva or tongue did not stain with each antibody, while an occasional reaction was observed in lymphocytes that infiltrated into the submucosal layer (Figure 4C

). LMO4- and/or LDB1-immunoreactive carcinoma cells were usually found at the periphery of the carcinoma cell nests. In addition, the reaction became more prominent in carcinoma cells at the invasive front (Figure 4A and B). Fibroblast-like cells and infiltrating lymphocytes surrounding carcinoma cells at the invasive front also exhibited nuclear staining. To quantify the immunoreactivity, we calculated the percentage of nuclear staining in carcinoma cells and compared this to the clinicopathological parameters of the samples. Immunoreactive carcinoma cells were observed in 18.69±22.89% (mean±1 s.d.) of LMO4 staining and 23.41±22.44 of LDB1 staining. However, the percentage of immunoreactive carcinoma cells was significantly increased in less-differentiated carcinoma tissues (*P*<0.01) (Table 1

**Table 1:** Association between LMO4 or LDB1 immunoreactivity and histological tumour differentiation

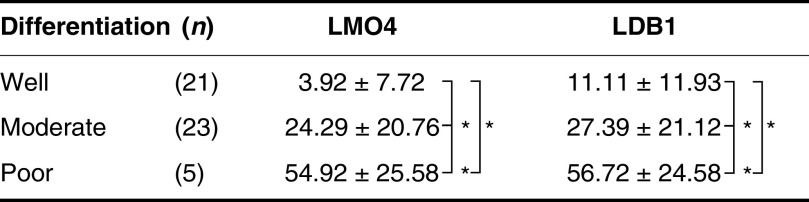		

**Figure 5:**
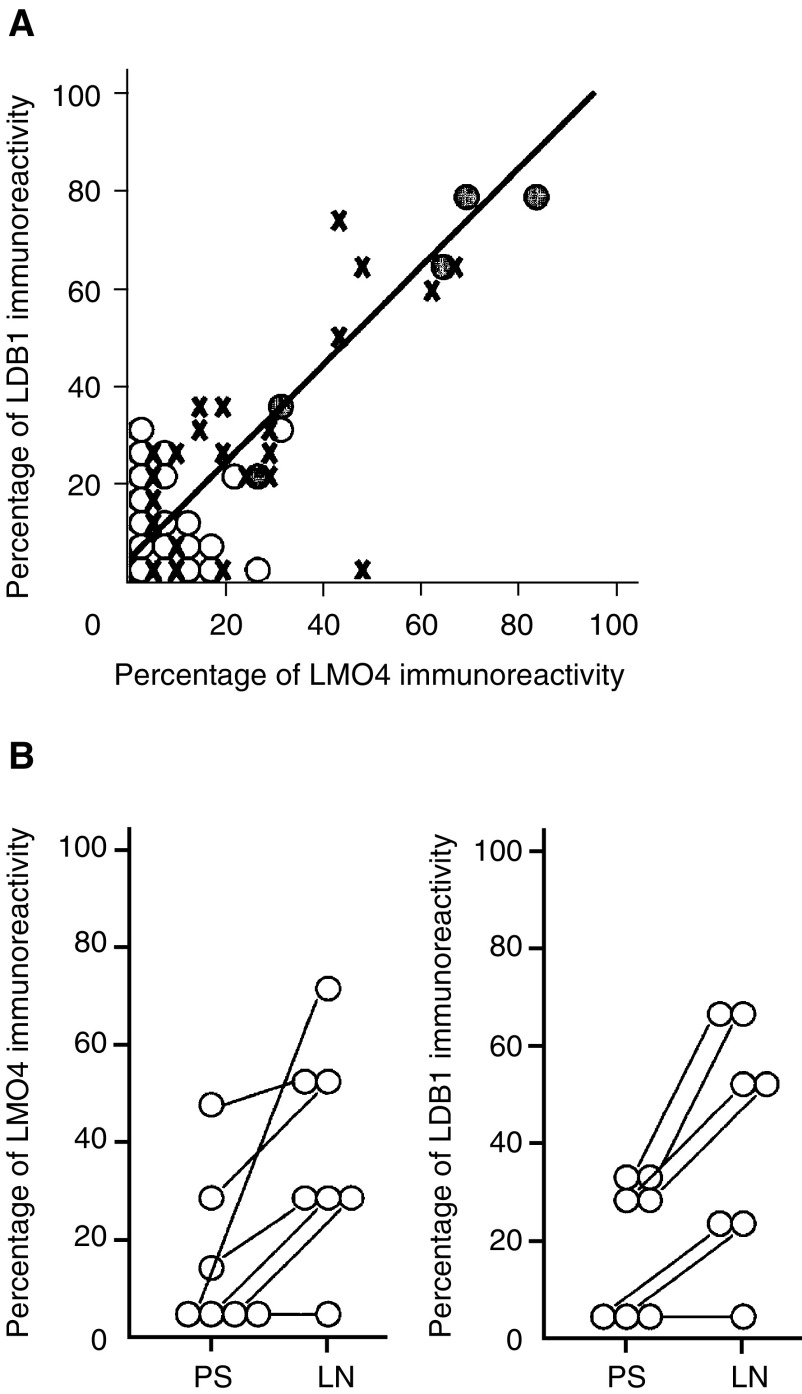
Immunoreactivity of LMO4 and LDB1 at the primary site of carcinomas (**A**) and in metastasised lymph nodes (**B**). (A) A positive direct correlation between LMO4 (horizontal line) and LDB1 (vertical line) immunoreactivity was found by simple linear regression (*r*^2^=0.669, *P*<0.01, *n*=49). Open circles, crossed, and shaded circles indicated well, moderately, and poorly differentiated squamous cell carcinomas, respectively. (B) The percentage of immunoreactive carcinoma cells in primary sites and metastasised cervical lymph nodes (LMO4; left, LDB1; right) was analysed by Wilcoxon's signed-rank test (*P*<0.05).

Percentage of immunoreactive carcinoma cells were counted and compared to histological differentiation grade by one-way analysis of variance and contrast statements (mean±1 s.d.). ^*^P<0.01.

) and their immunoreactivity of LMO4 and LDB1 showed a positive linear correlation (Figure 5A

). We also compared the immuno-reactivity between carcinoma cells at the primary site and metastasised lymph nodes. Carcinoma cells in the lymph nodes significantly increased the immunoreactivity of LMO4 (37.67±22.60) and LDB1 (38.07±23.84) compared to the corresponding primary sites (LMO4; 13.46±18.29, LDB1; 17.63±14.99) (Figure 5B). There was no statistical difference in the immunoreactivity with patient age, gender, and tumour size and grade (data not shown).

## DISCUSSION

Metastasis of squamous carcinoma cells to distant organs or lymph nodes requires several steps, including detachment from the primary site, dedifferentiation, invasion of the surrounding stroma and vessel walls, embolism, and stromal invasion and proliferation. Dedifferentiation of carcinoma cells initiates carcinoma progression toward a metastatic neoplasm (Vokes *et al*, 1993; Birchmeire *et al*, 1996; Lippman and Hong, 2001). Investigation of the molecular mechanism of carcinoma cell differentiation/dedifferentiation is a momentous subject in cancer cell biology and may contribute to the development of a novel strategy to relieve patient suffering from the disease. Although investigators reported that various factors are involved in carcinoma cell differentiation and dedifferentiation, accumulating evidence implies that transcriptional misregulation takes a critical part in the events (Angel *et al*, 1996; Hunter, 1997; Visvader *et al*, 1997; Battle *et al*, 2000; Cano *et al*, 2000). In the present study, our data demonstrate for the first time that LMO4 and LDB1 form protein complexes in the nucleus and are expressed in carcinoma cells at the invasive front, and their immunoreactivity is increased in less-differentiated carcinomas and in metastasised lymph nodes.

LMO4 and LDB1 are ubiquitously expressed in the mouse embryos, including epithelial and mesenchymal areas, when compared to the exclusive expression of other LMO family members and LDB2 in neuronal and haematopoietic cells (Visvader *et al*, 1997; Kenny *et al*, 1998; Sugihara *et al*, 1998; Toyama *et al*, 1998; Hermanson *et al*, 1999; Thaler *et al*, 2002). The LIM domain of LDBs contributes to the binding of transcription factors, including LIM-homeodomain, zinc-finger and basic helix–loop–helix (bHLH) proteins (Agulnick *et al*, 1996; Jurata *et al*, 1996; Bach *et al*, 1997; Morcillo *et al*, 1997). Formation of protein complexes synergistically activates the expression of target genes (Jurata *et al*, 1998; Milán *et al*, 1998). In the presence of LMO protein, it mediates the transcription factor binding to the LDB protein or competes with the direct binding between LDB and the transcription factor (Rabbitts, 1998; Thaler *et al*, 2002). These combinational actions are involved in the diversity of transcriptional regulation and specification of cell types (Visvader *et al*, 1997; Thaler *et al*, 2002). Misexpression of LMO1 and LMO2 by the chromosomal translocation is observed in T-cell leukaemia and facilitates the formation of an aberrant multimeric complex (Grütz *et al*, 1998; Rabbitts, 1998). Although the role in the pathology of carcinomas of epithelial origin is not clear, Visvader *et al* 2001 recently indicated that LMO4 and LDB1 are required to maintain the undifferentiation state of invasive breast carcinoma cells, and the forced expression of LMO4 inhibits differentiation of mammary epithelial cells. Increased expression of LMO4 and LDB1 in less-differentiated oral carcinomas represented in this study suggests an involvement in cellular dedifferentiation. Carcinoma cells located at the invasive front also exhibited an increase in immunoreactivity. Carcinoma cells located at the invasive front enhance the characteristics of epithelial–mesenchymal transition, which initiates invasion into the collagen matrices (Gobbert *et al*, 1985; Behrens *et al*, 1989; Frixen *et al*, 1991; Imai *et al*, 1995). Although the molecular mechanism of LMO4–LDB1 complex formation in carcinoma dedifferentiation is not clearly defined yet, LMO4 acts as a dominant negative by interacting with LDB1, thereby competing for binding between LDB1 and transcription factors. This dominant-negative effect of LMO4 inhibits differentiation of neuronal cells (Thaler *et al*, 2002). It is possible to speculate that increased expression of LMO4 may inhibit differentiation and accelerate invasion of oral carcinoma cells.

Another intriguing possibility comes from a study that GATA zinc-finger proteins interact with the LMO2–LDB1 complex and specifies a haematopoietic lineage differentiation (Wadman *et al*, 1997). However, overexpression of LMO2 in T-cell leukaemia results in the formation of a novel aberrant complex that substitutes a GATA protein to the E-box (CANNTG) binding bHLH and inhibits T-cell differentiation (Grütz *et al*, 1998). The *C. elegans* homologue of GATA, *Elt*, is a prerequisite for ectodermal cell differentiation (Gilleard and McGhee, 2001). GATA3 is expressed in the normal cervical squamous epithelial cells, but downregulated in progressive carcinoma cells (Steenbergen *et al*, 2002), suggesting a role of GATA in epithelial cell differentiation. The *cis*-acting E-box element is found in the E-cadherin promoter region and binding of the zinc-finger protein, SNAIL or SIP1, represses gene expression. E-cadherin has a central role in maintenance of the epithelial cell-type characteristic and the SNAIL/SIP1 inhibition of E-cadherin expression in squamous carcinoma cells accelerates dedifferentiation and invasion (Battle *et al*, 2000; Cano *et al*, 2000; Carver *et al*, 2001; Comijn *et al*, 2001). Our preliminary study showed significant expression of the *SNAIL* and *SIP1* genes in oral carcinomas (T Chiba and K Imai, manuscript in preparation). Although GATA expression in oral carcinomas is not yet known, it could be intriguing if misexpression of LMO4 results in substitution of GATA to SNAIL/SIP1 and inhibits carcinoma cell differentiation.

It is interesting to note that the carcinoma cells that metastasised to the cervical lymph nodes exhibited increased immunoreactivity to both LMO4 and LDB1 when compared to the corresponding primary sites of tumour. An increased reaction in the metastasised carcinoma cells suggests that LMO4- and LDB1-expressing carcinoma cells at the primary sites may ease the progress toward metastasis. It might also be plausible that the metastasised carcinoma cells upregulate LMO4 and LDB1 in the milieu of the lymph node. It is known that the local microenvironment modifies carcinoma cell differentiation (Aboseif *et al*, 1999; Liotta and Kohn, 2001). Further studies should be addressed to demonstrate a direct role for LMO4 and LDB1 in carcinoma metastasis.

The present study demonstrated that LMO4 and LDB1 form a protein complex and are overexpressed at the carcinoma invasive front, and in less-differentiated and metastasised squamous carcinoma cells. It suggests that misexpression of LMO4 and LDB1 expression may play a role in progression of neoplasm. Future avenues of research will clarify transcriptional partners and target genes of LMO4–LDB1 complexes, and elucidate the role of this pathway in the pathology of squamous cell carcinomas.
